# Supplementary material for: Diagnostic accuracy of contrast-enhanced CT for neck abscesses: A systematic review and meta-analysis of positive predictive value
Source: PLoS One. 2022 Oct 26;17(10):e0276544. doi: 10.1371/journal.pone.0276544 (PMC9604924; doi:10.1371/journal.pone.0276544)
Supplement: S1 Table — (DOCX) [file pone.0276544.s002.docx]

**S1 Table.** QUADAS-2 assessments.

| **Author** | **Patient selection** | **Index test** | **Reference standard** | **Flow and timing** | **Patient selection** | **Index test** | **Reference standard** |
| --- | --- | --- | --- | --- | --- | --- | --- |
| Chuang | Low | High | Low | High | Low | Low | Low |
| Wang | Low | Unclear | Low | Unclear | Low | Low | Low |
| Côrte | Low | Unclear | Low | Unclear | High | Low | Low |
| Boscolo-Rizzo | Low | Low | Low | High | High | Low | Low |
| Page | Low | Unclear | Low | Unclear | High | Low | Low |
| Elden | Low | Unclear | Low | High | High | Low | Low |
| Seer Yee | Low | Low | Low | Unclear | Low | Low | Low |
| Collins | Low | Unclear | Low | High | High | Low | Low |
| Freling | Low | Unclear | Low | Unclear | Low | Low | Low |
| Meyer | Low | Low | Low | Unclear | High | Low | Low |
| Kirse | Low | High | Low | Unclear | High | Low | Low |
| Hoffman | Low | Unclear | Low | Unclear | High | Low | Low |
| Choi | Low | Low | Low | Unclear | High | Low | Low |
| Ban | Low | Low | Low | High | Low | Low | Low |
| Wong | Low | Unclear | Low | High | High | Low | Low |
| Lazor | Low | Unclear | Low | Low | Low | Low | Low |
| Malloy | Low | Unclear | Low | Unclear | High | Low | Low |
| Smith | Low | Unclear | Low | Low | Low | Low | Low |
| Miller | Low | Low | Low | Low | High | Low | Low |
| Saluja | Low | Low | Low | Low | High | Low | Low |
| Stone | Low | Low | Low | Low | High | Low | Low |
| Vural | Low | Unclear | Low | High | High | Low | Low |
| Kurzyna | Low | Unclear | Low | High | High | Low | Low |
