# Supplementary material for: Diagnostic accuracy of contrast-enhanced CT for neck abscesses: A systematic review and meta-analysis of positive predictive value
Source: PLoS One. 2022 Oct 26;17(10):e0276544. doi: 10.1371/journal.pone.0276544 (PMC9604924; doi:10.1371/journal.pone.0276544)
Supplement: S2 Table — (DOCX) [file pone.0276544.s003.docx]

**S2 Table.** Studies excluded due to small sample size.

| **First author** | **Year** | **N** | **Design** | **Patients** | **Location** | **TP** | **FP** | **PPV** |
| --- | --- | --- | --- | --- | --- | --- | --- | --- |
| Nagy | 1997 | 20 | retrospective | children | multiple | 19 | 1 | 0.950 |
| Daya | 2005 | 19 | retrospective | children | multiple | 17 | 2 | 0.895 |
| Joshua | 2012 | 15 | retrospective | children | multiple | 14 | 1 | 0.933 |
| Rosenthal | 2011 | 17 | retrospective | all ages | maxillofacial | 16 | 1 | 0.941 |
| Grisaru-Soen | 2010 | 17 | retrospective | children | multiple | 13 | 4 | 0.765 |
| Wetmore | 1998 | 13 | retrospective | children | multiple | 12 | 1 | 0.923 |
| Ungkanont | 1995 | 12 | retrospective | children | multiple | 10 | 2 | 0.833 |
| Flanary | 1997 | 12 | retrospective | children | multiple | 10 | 2 | 0.833 |
| Patel | 1992 | 11 | prospective | not reported | PTA | 11 | 0 | 1.000 |
| Bolton | 2013 | 10 | retrospective | children | multiple | 10 | 0 | 1.000 |
| Boucher | 1999 | 10 | retrospective | children | RPA | 4 | 6 | 0.400 |
| Scott | 1999 | 10 | prospective | all ages | PTA | 9 | 1 | 0.900 |
| Velhonoja | 2021 | 9 | retrospective | children | multiple | 8 | 1 | 0.889 |
| Courtney | 2007 | 8 | retrospective | children | RPA | 6 | 2 | 0.750 |
| Varelas | 2019 | 6 | retrospective | children | PTA | 6 | 0 | 1.000 |
| Courtney | 2007 | 6 | retrospective | children | RPA | 5 | 1 | 0.833 |
| Ravindranath | 1993 | 5 | retrospective | children | RPA | 5 | 0 | 1.000 |
| Sichel | 2006 | 5 | retrospective | children | PPA | 3 | 2 | 0.600 |
| McClay | 2003 | 1 | retrospective | children | multiple | 1 | 0 | 1.000 |

N, number of patients who had abscess on CT and surgery; PPA, parapharyngeal abscess; PTA, peritonsillar abscess; RPA, retropharyngeal abscess; TP, true positive; FP, false positive; PPV, positive predictive value; Se, sensitivity; Sp, specificity.
