# Supplementary material for: Diagnostic accuracy of contrast-enhanced CT for neck abscesses: A systematic review and meta-analysis of positive predictive value
Source: PLoS One. 2022 Oct 26;17(10):e0276544. doi: 10.1371/journal.pone.0276544 (PMC9604924; doi:10.1371/journal.pone.0276544)
Supplement: S3 Table — (DOCX) [file pone.0276544.s004.docx]

**S3 Table.** Technical details of CT acquisition in the included studies.

| **First author** | **Manufacturer & model** | **Number of slices** | **Slice thickness (mm)** | **Contrast injection volume (ml)** | **Saline volume (ml)** | **Contrast injection rate (ml/sec)** | **Delay (s)** |
| --- | --- | --- | --- | --- | --- | --- | --- |
| Chuang | GE Lightspeed | 16 | 3.75 | 100 for adults, 1.5/kg for children | - | 2 for adults, 1.5 for children | 60 |
| Wang | - | - | - | - | - | - | - |
| Côrte | - | - | - | - | - | - | - |
| Boscolo-Rizzo | - | - | 3 | - | - | - | - |
| Page | - | - | - | - | - | - | - |
| Elden | GE 9800 | - | - | - | - | - | - |
| Seer Yee | Toshiba Aquilion Model TSX-101A 2002 | 4 | 2 | 50 for adults, 1.5-2/kg for children | 30 | 2 | - |
| Collins | - | - | - | - | - | - | - |
| Freling | Philips MX-8000 | - | 3.2 | 80 for adults, 2/kg for children | - | 2 for adults, 1.5-2 for children | 60 |
| Meyer | - | - | - | - | - | - | - |
| Kirse | - | - | - | - | - | - | - |
| Hoffman | - | - | - | - | - | - | - |
| Choi | - | - | - | - | - | - | - |
| Ban | - | - | - | - | - | - | - |
| Wong | - | - | - | - | - | - | - |
| Lazor | Siemens DR 3 | - | - | - | - | - | - |
| Malloy | - | - | - | - | - | - | - |
| Smith | - | - | - | - | - | - | - |
| Miller | GE CTi | - | 5 | 100 | - | - | - |
| Saluja | - | - | - | - | - | - | - |
| Stone | - | - | - | - | - | - | - |
| Vural | - | - | - | - | - | - | - |
| Kurzyna | - | - | - | - | - | - | - |

“-“ denotes missing data.
